# Isolation of equine multipotent mesenchymal stromal cells by enzymatic tissue digestion or explant technique: comparison of cellular properties

**DOI:** 10.1186/1746-6148-9-221

**Published:** 2013-10-29

**Authors:** Claudia Gittel, Walter Brehm, Janina Burk, Henriette Juelke, Carsten Staszyk, Iris Ribitsch

**Affiliations:** 1 Large Animal Clinic for Surgery, University of Leipzig, An den Tierkliniken 21, 04103 Leipzig, Germany; 2 Translational Centre for Regenerative Medicine (TRM), University of Leipzig, Philipp-Rosenthal-Straße 55, 04103 Leipzig, Germany; 3 Department of Veterinary-Anatomy, -Histology and -Embryology, Faculty for Veterinary Medicine, Justus-Liebig-University Giessen, Frankfurter Str. 98, 35392 Giessen, Germany; 4 Equine Hospital, Clinic for Equine Surgery, University of Veterinary Medicine, Veterinärplatz 1, 1210 Vienna, Austria

**Keywords:** Horse, Regenerative medicine, Collagenase, Cell isolation, Scleraxis

## Abstract

**Background:**

The treatment of tendon lesions with multipotent mesenchymal stromal cells (MSCs) is widely used in equine medicine. Cell sources of MSCs include bone marrow, as well as solid tissues such as adipose tissue. MSCs can be isolated from these solid tissues either by enzymatic digestion or by explant technique. However, the different preparation techniques may potentially influence the properties of the isolated MSCs. Therefore, the aim of this study was to investigate and compare the effects of these two different methods used to isolate MSCs from solid tissues.

Equine adipose tissue, tendon and umbilical cord matrix served as solid tissue sources of MSCs with different stiffness and density. Subsequent to tissue harvest, MSCs were isolated either by enzymatic digestion with collagenase or by explant technique. Cell yield, growth, differentiation potential and tendon marker expression were analysed.

**Results:**

At first passage, the MSC yield was significantly higher in enzymatically digested tissue samples than in explanted tissue samples, despite a shorter period of time in primary culture. Further analysis of cell proliferation, migration and differentiation revealed no significant differences between MSCs isolated by enzymatic digestion and MSCs isolated by explant technique. Interestingly, analysis of gene expression of tendon markers revealed a significantly higher expression level of scleraxis in MSCs isolated by enzymatic digestion.

**Conclusions:**

Both isolation techniques are feasible methods for successful isolation of MSCs from solid tissues, with no major effects on cellular proliferation, migration or differentiation characteristics. However, higher MSC yields were achieved in a shorter period of time by collagenase digestion, which is advantageous for the therapeutic use of MSCs. Moreover, based on the higher level of expression of scleraxis in MSCs isolated by enzymatic digestion, these cells might be a better choice when attempting tendon regeneration.

## Background

Multipotent mesenchymal stromal cells (MSCs) are described as highly proliferative cells with the capacities of tri-lineage differentiation and plastic adherence [1,2]. These cells are a promising cell population for alternative treatments of orthopaedic injuries. In equine athletes, MSCs are frequently applied to treat tendon injuries, such as core lesions in the superficial digital flexor tendon (SDFT). Clinical studies have shown more favourable outcomes for this treatment as compared to conventional treatment [3-8].

Currently, the most widely used tissue sources for isolation of MSCs in equine medicine are bone marrow (BM) and subcutaneous adipose tissue (AT) [6,9,10].

Although recovery of MSCs from BM is common, there are concerns about the invasive BM aspiration procedure and the potential complications for donor horses [11,12]. Furthermore, there are cell culture-specific restrictions associated with MSCs derived from BM, such as early cell senescence associated with donor age and limited recovery of MSCs [12-14]. In comparison to BM, various solid tissues, such as AT, tendon tissue or umbilical cord matrix (UCM) appear to yield higher numbers of MSCs that are highly proliferative and that also possess tri-lineage differentiation potential [15-18].

For clinical use, reliably repeatable isolation of an adequate number of MSCs is of great importance [19,20]. Different protocols are available for the isolation of MSCs from solid tissues [9,21]. However, the potential impact of the choice of protocol on cell yield and characteristics of equine MSCs has not yet been investigated.

The most frequently used method for isolation of MSCs from solid tissue is digestion by proteolytic enzymes, such as collagenase [22-26]. After digestion, the nucleated cell fraction is released and can be seeded onto plastic culture dishes, where MSCs adhere and thus can be separated from the remaining non-adherent cells.

Other studies have described the isolation of MSCs from solid tissues by a method referred to as the explant technique [27-29]. For this technique, excised tissue is cut into small pieces and plated onto plastic culture dishes. MSCs migrate from the pieces of tissue and adhere to the plastic surface. This method requires less labour and is less invasive to the cells. Moreover, it appears to have less impact on cell viability [28] and might be advantageous due to the initial presence of native tissue and similar physical environment [30].

Enzymatic digestion may negatively affect cellular properties, due to the major alteration of the natural environment of the cells [30], considering that differences in culture conditions also cause alterations of MSC properties [31-33]. However, the impact of differences in the isolation method on MSC characteristics is not yet completely understood [15,19,28,34,35].

In this study, we isolated MSCs from equine solid tissues by enzymatic digestion or by explant technique. We subsequently compared cell yield, proliferation, migration and differentiation potential of the isolated cells, as well as tendon marker expression, in order to investigate the influence of the isolation technique on characteristics of isolated equine MSCs. For this purpose, we used three types of solid tissues as cell sources for the experiments (AT, SDFT and UCM). All three of these tissues are of different density and stiffness and are known to host MSCs.

## Methods

### Tissue collection

Equine AT, SDFT and UCM were used as tissue sources for MSC isolation. Subcutaneous AT and SDFT, respectively, were harvested from eight adult horses (mean age: 3.5 years, interquartile range (IQR): 1.75) following euthanasia. UCM samples were collected from 14 foals immediately after birth. Sampling procedures followed the applicable regulations of animal welfare and were approved by the local ethics committee (Landesdirektion Leipzig, A 13/10).

For subcutaneous AT collection, the paraxial caudodorsal gluteal region was clipped and the skin was aseptically prepared. An incision of approximately 10 cm length was made in the skin, and approximately 15 g of subcutaneous AT was obtained with a scalpel and forceps. The tissue was processed immediately.

Tendon samples were obtained from the SDFT of one forelimb of each horse. The palmar region between carpus and fetlock was clipped and the skin was aseptically prepared. After a skin incision of approximately 10 cm length was made, about 15 g of tendon tissue was recovered with a scalpel and forceps and processed immediately.

For UCM collection, approximately 15 cm of the umbilical cord was recovered immediately after foal birth. Umbilical cord tissue was washed with povidone-iodine solution (Braun, Melsungen, Germany) and 70% ethanol (apomix, Halle/Salle, Germany) for disinfection. The umbilical cord was placed in a sterile container with 150 ml phosphate-buffered saline (PBS; PAA, Cölbe, Germany), 0.1% gentamicin (PAA) and 2.5 μg/ml amphotericin B (Life Technologies GmbH, Darmstadt, Germany) and stored overnight at room temperature.

### Tissue preparation and cell isolation

Following tissue recovery, the samples were processed under sterile conditions. Blood vessels were dissected from UCM samples prior to further preparation.

Equal amounts, of approximately 6 g, of each specimen were subjected to cell isolation either by tissue digestion or by explant technique.

For digestion, samples of AT, SDFT and UCM were cut into pieces of 0.1-0.2 cm size and washed with Hank’s balanced salt solution (HBSS; Life Technologies GmbH). Subsequently, the minced tissue pieces were placed in plastic tubes (BD, Bioscience, Heidelberg, Germany) containing HBSS and collagenase I (Life Technologies GmbH, catalogue number 17100017) and were incubated at 37°C in a continuously shaking water bath. AT was digested for 4 hours in a collagenase I solution at a concentration of 0.8 mg/ml. SDFT was digested for 6 hours at a collagenase concentration of 5.6 mg/ml. UCM was digested for 6 hours at a collagenase concentration of 2.4 mg/ml. After incubation in collagenase solution, remaining tissue pieces were discarded. The digestion solution was filtered with a cell filter (pore size 70 μM; BD Bioscience). The mononuclear cell (MNC) fraction obtained was subjected to two cycles of centrifugation (437 *g*, 5 min, 4°C) and washing in PBS. Subsequently, MNCs were counted using a microscope counting chamber. The cell pellet was resuspended in standard cell culture medium consisting of low glucose (1 g/l) Dulbecco’s Modified Eagle Medium (DMEM; Life Technologies GmbH) supplemented with 20% foetal calf serum (FCS; Sigma-Aldrich, Hamburg, Germany, catalogue number F7524), 0.1% gentamicin, and 1% penicillin-streptomycin (PAA). UCM cell culture medium was additionally supplemented with 0.5 μg/ml amphotericin B until first passage to prevent fungal contamination of the cultures [14]. MNCs were seeded onto plastic culture dishes (BD Bioscience) at a density of approximately 20,000 cells/cm^2^. Primary cultures (passage [P] 0) were cultivated under standard culture conditions, i.e. humidified atmosphere at 37°C and 5% CO_2,_ and the culture medium was changed twice a week. MSCs obtained by digestion, hereafter referred to as “di-MSCs,” were passaged by trypsinisation (Trypsin, Life Technologies GmbH) when the cell colonies reached confluency.

**Figure 1:**
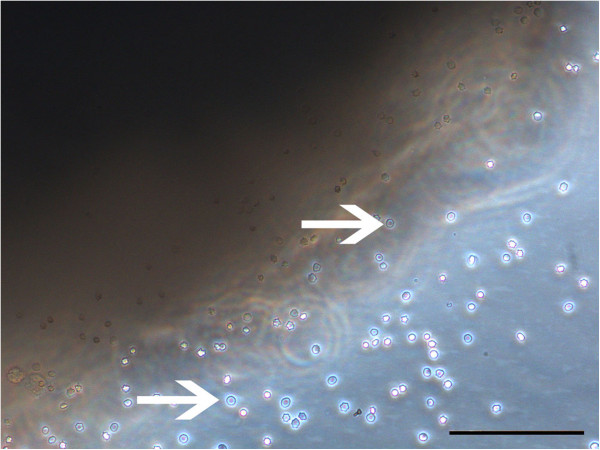
**Explant technique.** Single cells (white arrows) migrated from the margin of the tissue piece (tendon) and adhered onto the plastic surface to form cell colonies. Following several days in culture, cells developed typical spindle-shaped morphology. Scale bar = 100 μm.

For the isolation of MSCs by explant technique, solid tissues were dissected into pieces of approximately 0.5 × 0.5 × 0.5 cm size using a surgical blade and forceps and then washed in PBS. Tissue pieces were placed onto cell culture dishes (TPP, Trasadingen, Switzerland) and covered with standard cell culture medium to allow cell migration from the tissue pieces onto the culture plate (Figure 1). Culture conditions were identical to those following enzymatic digestion. After 7 days, tissue pieces were carefully removed. Primary cultures (P0) were passaged at confluency of colonies to obtain MSCs isolated by explant technique, hereafter referred to as “ex-MSCs”.

In the subsequent assays, di-MSCs and ex-MSCs were compared separately for each tissue type to assess the potential effects of the two different isolation techniques. For these assays, seven paired di- and ex-MSC samples derived from adipose and tendon tissue, respectively, were used. Due to a partial contamination of UCM samples, 10 unpaired di- and ex-MSC samples derived from UCM were available for the following assays.

### MSC yield

The number of MSCs was counted following trypsinisation at the first cell harvest at confluency of colonies. The yield of MSCs per gram of tissue per primary culture days was calculated according to the following formula;

$$\mathrm{MSC}\;\mathrm{yield}=\frac{\text{cell}\;\text{number}\;\text{at}\;\mathrm{first}\;\text{cell}\;\mathrm{harvest}}{\left[\mathrm{tissue}\;\mathrm{weight}\right]\times \left[\text{number}\;\mathrm{of}\;\mathrm{primary}\;\mathrm{culture}\;\mathrm{days}\right]}.$$

### Proliferation assays

From P1 to P7, cells were plated in culture flasks (BD Bioscience) at a density of 3,000 MSCs/cm^2^ and incubated to subconfluency in standard cell culture medium under standard culture conditions. Subsequently, MSCs were trypsinised, cell numbers were determined and cells were subjected to seeding as described above. Generation times (GTs) were calculated separately for each passage based on cell counts and culture time according to the following formula:

$$\mathrm{GT}=\frac{\text{cell}\;\text{culture}\;\text{time}}{\text{population}\;\text{doubling}}$$

$$\text{Population}\;\text{doubling}=\frac{\ln\left(\frac{\text{cell}\;\text{number}\;\text{at}\;\text{harvest}}{\text{cell}\;\text{number}\;\text{at}\;\text{plating}}\right)}{\ln2}$$

Cell proliferation was additionally assessed in P3, as well as in P8, by determining the relative increase in the number of metabolically active cells using a 3-(4,5-dimethylthiazol-2-yl)-5-(3-carboxymethoxyphenyl)-2-(4-sulfophenyl)-2H-tetrazolium (MTS) proliferation assay, performed according to the manufacturer’s instructions. Briefly, 1,000 MSCs per well were seeded onto a 96-well plate and incubated under standard culture conditions. At day 1 CellTiter 96® AQ_ueous_ One Solution Reagent (Promega, Mannheim, Germany) was added to the medium and samples were incubated at 37°C, 5% CO_2_ for 4 hours. Subsequently, the absorbance at 490 nm was measured using Tecan Safire™ (Magellan™ Software; Tecan Group Ltd., Maennedorf, Switzerland). The same steps were performed in another assay at day 7 after seeding. Proliferation rates (PRs) were calculated using the following formula:

$$\mathrm{PR}=\frac{\text{mean}\;\text{optical}\;\text{density}\;\text{at}\;\text{day}\;7}{\text{mean}\;\text{optical}\;\text{density}\;\text{at}\;\text{day}\;1}.$$

### Migration potential

**Figure 2:**
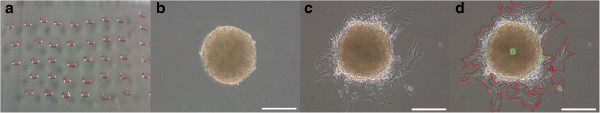
**Migration assay. (a)** Culture of MSCs using a spheroid system. **(b)** Attachment of spheroid 4 hours after seeding **(c)** and MSC migration 24 hours after spheroid seeding. **(d)** At the 24 hour time point, the spheroid size was measured (area B) and subtracted from the area of the maximum range of migrated MSCs (area A). Scale bar = 200 μm. (MSCs: multipotent mesenchymal stromal cells).

The migration potential of MSCs was determined in P3 by spheroid culture. 5,000 cells per spheroid were cultivated in hanging drops using non-adherent dishes (Greiner Bio-One GmbH, Frickenhausen, Germany) and standard cell culture medium supplemented with methylcellulose (Sigma-Aldrich) (Figure 2a). After 24 hours, spheroids were harvested by rinsing with PBS. The spheroids obtained were plated in standard cell culture medium on adherent 6-well plates (BD Bioscience) and incubated under standard culture conditions to allow the MSCs to migrate out of the spheroids (Figure 2b,c). Photographs (IX51 research microscope; CC-12 digital colour camera; Cell^A software; Olympus Soft Imaging Solutions GmbH, Muenster, Germany) of spheroids and migrating MSCs were taken after 24 hours of incubation (Figure 2d). The migration area was determined following measurement of spheroid size and the area covered by MSCs.

$$\text{migration}\;\text{area}=\left(\text{area}\;\text{covered}\;\text{by}\;\text{MSCs}\right)–\left(\text{spheroid}\;\text{size}\right)$$

### *In vitro* differentiation assays

#### Adipogenesis assay

1,500 cells/cm^2^ in P3 were plated onto adherent 12-well plates (BD Bioscience) in standard cell culture medium to allow cell attachment. After 3 days, adipogenic differentiation was induced by replacement of culture medium with adipogenic differentiation medium consisting of DMEM F-12 (PAA), 15% rabbit serum, 1 μM dexamethasone, 100 μM indomethacin, 500 μM 3-isobutyl-1-methylxanthine, 700 nM bovine insulin (all Sigma-Aldrich) and antibiotics (0.1% gentamicin, 1% penicillin-streptomycin), which had been evaluated previously [36]. After 3 days of incubation, MSCs were fixed with 50% ethanol (Roth, Karlsruhe, Germany) and stored at -20°C until further processing. Cell staining was performed with oil red O solution (Sigma-Aldrich). Two random photographs (IX51 research microscope; CC-12 digital colour camera; Cell^A software) of each well were evaluated by two blinded observers using a scoring system based on the percentage of differentiated cells and the size of intracellular lipid vacuoles (Table 1).

**Table 1:** Semiquantitative adipogenic differentiation score

**% of differentiated cells among all MSCs in field of view (10× magnification)**		**Size and arrangement of lipid droplets**	
0 points	0 – 5%	0 points	No lipid droplets
1 point	>5 – 50%	1 point	Predominantly few isolated and small-sized (< 1/3 of nucleus diameter) lipid droplets
2 points	>50 – 80%	2 points	Predominantly medium-sized (approximately 1/3 of nucleus diameter) lipid droplets, surrounding the nucleus
3 points	>80 - 100%	3 points	Predominantly large-sized (> 1/3 of nucleus diameter) lipid droplets, merging around the nucleus

Two criteria scoring system for semiquantitative evaluation of adipogenic differentiation following the oil red O staining procedure. A maximum score of 6 points was possible, with a maximum of 3 points for each criterion.

#### Osteogenesis assay

500 cells/cm^2^ in P3 were plated onto adherent 12-well plates in standard cell culture medium. After 3 days of cell attachment, the culture medium was removed and cells were incubated with osteogenic differentiation medium consisting of DMEM F-12, 10% FCS, 0.1 mM L-ascorbate-2-phosphate, 0.1 μM dexamethasone, 10 mM β-glycerophosphate (all Sigma-Aldrich) and antibiotics (0.1% gentamicin, 1% penicillin-streptomycin). Following incubation for 21 and 35 days, cells were fixed with 4% paraformaldehyde (Roth) and stored at -20°C until further processing. For detection of osteogenic differentiation, we used von Kossa staining of extracellular calcium-deposits. Samples with qualitative evidence of differentiation were further assessed as previously described [37]. Briefly, absorbance at 492 nm was determined for stained differentiated samples and undifferentiated controls (Tecan Safire™, Magellan™ Software). Osteogenic differentiation at day 21 and day 35 was quantified by the index of osteogenic differentiation (IOD):

$$\mathrm{IOD}=\frac{\text{optical}\;\text{density}\;\mathrm{of}\;\mathrm{differentiated}\;\mathrm{samples}}{\text{optical}\;\text{density}\;\mathrm{of}\;\mathrm{controls}}$$

#### Chondrogenesis assay

Chondrogenic differentiation of P3 MSCs was performed in a 3D-pellet culture system. To obtain stable 3D-cell pellets, 500,000 cells per assay were placed into a 15 ml polypropylene centrifuge tube (BD Bioscience) and centrifuged for 5 minutes at 240 *g*. The cell pellets were incubated under standard culture conditions with chondrogenic differentiation medium consisting of high glucose DMEM (4.5 g/l; PAA), 10 ng/ml TGF-β (Acris Antibodies, Herford, Germany), 1% ITS + premix (BD Bioscience), 100 μM L-ascorbate-2-phosphate, 100 nM dexamethasone (both Sigma-Aldrich), 400 nM proline (Roth) and antibiotics (0.1% gentamicin, 1% penicillin-streptomycin). Pellet culture was terminated after 21 days by fixation with 4% paraformaldehyde. Subsequently, the pellets were embedded in paraffin and 6 μm paraffin sections were prepared for Alcian Blue, Masson’s Trichrome and Safranin O staining. Pellets showing qualitative evidence of chondrogenic differentiation by Alcian Blue and Masson’s Trichrome staining were then semiquantitatively evaluated using the Bern Score [38] based on the Safranin O staining.

### Gene expression analysis of tendon markers

Total RNA was isolated from MSC monolayer cultures (P3) using the RNeasy Mini Kit with On-Column DNase digestion (both Qiagen, Hilden, Germany). All steps were performed according to the manufacturer’s instructions. RNA was quantified (NanoDrop 1000 Spectrophotometer; NanoDrop Software; Thermo Fisher Scientific, Wilmington, DE, USA) and 1,000 ng of RNA was converted to first strand cDNA with Omniscript Reverse Transcriptase (Qiagen). Fluorescence-based real-time quantitative PCR (qPCR) was performed and monitored using a 7500 Real-Time PCR System (Applied Biosystems, Darmstadt, Germany). Targeted genes included the tendon markers collagen 1A2 and scleraxis. Each cDNA sample was mixed with iQ SYBR Green Supermix (Bio-Rad Laboratories, Munich, Germany) and gene-specific forward and reverse primers (primer details are shown in Table 2), and the threshold cycle was determined for each sample. Cycling conditions were 40 cycles of denaturation (90°C for 30 sec), annealing (60°C for 30 sec) and elongation (72°C for 30 sec). A set of negative controls was processed in the same manner except that cDNA was replaced with water. The relative copy numbers of target genes were calculated from the standard curve for each gene and normalised to the housekeeping gene, glyceraldehyde 3-phosphate dehydrogenase (GAPDH).

**Table 2:** Gene primer sequences used for qPCR

**Gene**	**Primer sequence**	**Amplicon size**
GAPDH	F: CCAGAACATCATCCCTGCTT	158
NM_001163856		
	R: CGTATTTGGCAGCTTTCTCC	
Collagen 1A2	F: GAAGATGGTCACCCTGGAAA	177
XM_001492939		
	R: AGGTTCACCCTTCACACCTG	
Scleraxis	F: ACAGAAAGACGGCGATTCGGAGTT	207
NM_001105150		
	R: AAAGTTCCAGTGGGTCTGGGCAA	

List of genes analysed by real-time quantitative PCR. GenBank accession numbers of the sequences used for primer design with Primer3 free online software as well as primer sequences and length of the amplicons in base pairs are shown. The sequences of primers of the gene scleraxis were kindly provided by the Institute of Anatomy, University of Veterinary Medicine, Foundation, Hannover, Germany. (F: forward, R: reverse).

### Statistical analysis

The data were processed using PASW Statistics 18 (IBM Deutschland GmbH, Ehningen, Germany). Comparisons were made between di-MSCs and ex-MSCs, for each tissue type separately. The Wilcoxon signed-rank test was used for MSCs derived from AT and from SDFT. Comparison of di-MSCs and ex-MSCs derived from UCM was performed with the Mann–Whitney *U* test. Significance was set at a value of p ≤ 0.05. Data were reported as median (IQR). Outliers were included in the data analysis. Mild outliers are any data values that lie between 1.5 and 3.0 times and extreme outliers are any data values that lie more than 3.0 times the IQR below the first quartile or above the third quartile.

## Results

### MSC yield

MSCs were successfully obtained from all three tissues (AT, SDFT and UCM) using both methods, enzymatic digestion and explant technique.

The MSC yield per tissue gram per day, following primary culture was significantly higher using the digestion method in all tissues (Table 3). Furthermore, ex-MSCs required significantly more days in primary culture until the first cell harvest was possible.

**Table 3:** MSC yield per gram per days for each tissue type

	**AT-MSC**		
	**di-MSC**	**ex-MSC**	
Days in primary culture	6 (2)	11 (5)	*
MSC yield per gram	21.13 × 10^5^ (18.00 × 10^5^)	0.79 × 10^5^ (1.36 × 10^5^)	*
MSC yield per gram per days	352.2 × 10^3^ (180.9 × 10^3^)	13.2 × 10^3^ (12.7 × 10^3^)	*
	**SDFT-MSC**		
	**di-MSC**	**ex-MSC**	
Days in primary culture	7 (2)	10.5 (1)	*
MSC yield per gram	17.49 × 10^5^ (10.72 × 10^5^)	1.62 × 105 (2.05 × 10^5^)	*
MSC yield per gram per days	291.5 × 10^3^ (176.4 × 10^3^)	17.3 × 10^3^ (19.6 × 10^3^)	*
	**UCM-MSC**		
	**di-MSC**	**ex-MSC**	
Days in primary culture	10 (4.25)	18 (4)	*
MSC yield per gram	4.16 × 10^5^ (12.66 × 10^5^)	0.70 × 10^5^ (0.66 × 10^5^)	*
MSC yield per gram per days	61.7 × 10^3^ (134.2 × 10^3^)	4.2 × 10^3^ (4.1 × 10^3^)	*

Data are presented as median (IQR).

(AT-MSC: adipose tissue-derived MSC; di-MSC: MSC isolated by digestion method; ex-MSC: MSC isolated by explant technique; IQR: interquartile range; MSC: multipotent mesenchymal stromal cell; SDFT-MSC: tendon-derived MSC; UCM-MSC: umbilical cord matrix-derived MSC).

*Indicates significance with a p-value ≤ 0.05.

### Proliferation assays

There were no significant differences in proliferation between di-MSCs and ex-MSCs (p > 0.05). However, we did observe trends in proliferation that were dependent on the respective tissue source.

**Figure 3:**
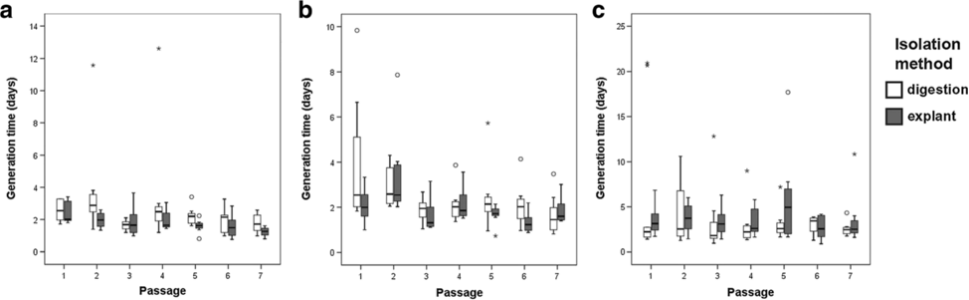
**Generation times of MSCs from passages 1 to 7 for each tissue type.** Circle indicates mild outlier; asterisk indicates extreme outlier. No significant differences were observed between di-MSCs and ex-MSCs for each tissue type (p-values > 0.05). Generation time = (cell culture time)/(population doubling). **(a)** (AT-MSC: adipose tissue-derived MSC; MSC: multipotent mesenchymal stromal cell; **(b)** SDFT-MSC: tendon-derived MSC; **(c)** UCM-MSC: umbilical cord matrix-derived MSC).

Ex-MSCs derived from AT and SDFT had lower GTs in comparison to the respective di-MSCs (Figure 3a and Figure 3b), indicating faster PRs of ex-MSCs from these tissues. In contrast, ex-MSCs derived from UCM had higher GTs than the respective di-MSCs in most passages (Figure 3c), indicating that in UCM tissue, di-MSCs proliferated faster.

**Figure 4:**
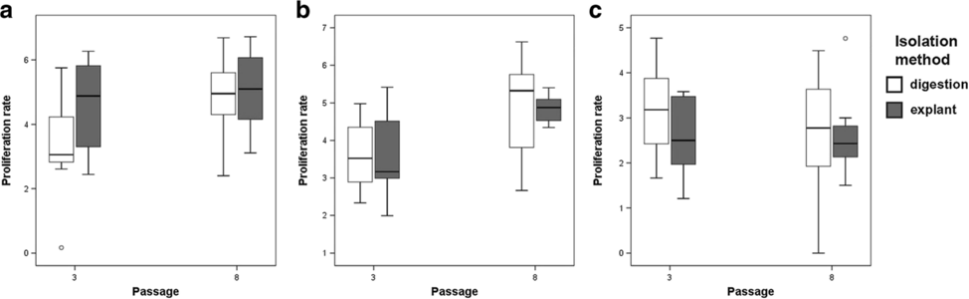
**Proliferation rates of MSCs (passages 3 and 8) for each tissue type.** Circle indicates mild outlier. No significant differences were observed between di-MSCs and ex-MSCs for each tissue type (p-values > 0.05). **(a)** (AT-MSC: adipose tissue-derived MSC; MSC: multipotent mesenchymal stromal cell; **(b)** SDFT-MSC: tendon-derived MSC; **(c)** UCM-MSC: umbilical cord matrix-derived MSC).

The results from the MTS proliferation assay supported these findings in MSCs from AT and UCM, as higher PRs were found in ex-MSCs from AT and lower PRs in ex-MSCs from UCM, in early as well as late passages (Figure 4a and Figure 4c). However, the finding that ex-MSCs derived from SDFT proliferated faster than the di-MSCs could not be confirmed by the MTS assay, in which di-MSCs displayed higher PRs (Figure 4b).

### Migration potential

Di-MSCs showed a higher migration potential in comparison to the corresponding ex-MSC samples, regardless of the tissue type (Table 4). However, this difference was not significant.

**Table 4:** Migration and differentiation potential of MSCs

	**AT-MSC**	
	**di-MSC**	**ex-MSC**
Migration area [mm^2^]	0.237 (0.033)	0.207 (0.384)
Adipogenic differentiation score	5.5 (0.0)	5.5 (0.5)
IOD (day 21)	2.2418 (0.6207)	2.0118 (1.5715)
IOD (day 35)	3.7193 (1.5035)	4.8596 (3.3090)
Chondrogenic differentiation score	2.5 (0.75)	3.0 (0.5)
	**SDFT-MSC**	
	**di-MSC**	**ex-MSC**
Migration area [mm^2^]	0.353 (0.213)	0.238 (0.126)
Adipogenic differentiation score	5.5 (0.0)	5.5 (0.0)
IOD (day 21)	2.8386 (1.2352)	3.0644 (1.7268)
IOD (day 35)	4.2629 (1.4771)	3.7185 (1.8768)
Chondrogenic differentiation score	1.875 (2.75)	3.5 (2.5)
	**UCM-MSC**	
	**di-MSC**	**ex-MSC**
Migration area [mm^2^]	0.090 (0.124)	0.048 (0.128)
Adipogenic differentiation score	4.5 (1.0)	4.25 (0.75)
IOD (day 21)	1.0211 (0.2223)	1.1055 (0.2843)
IOD (day 35)	1.2135 (0.2767)	0.9519 (0.0800)
Chondrogenic differentiation score	3.5 (1.5)	3.0 (2.75)

Data are presented as median (IQR).

(AT-MSC: adipose tissue-derived MSC; di-MSC: MSC isolated by digestion method; ex-MSC: MSC isolated by explant technique; IOD: index of osteogenic differentiation; IQR: interquartile range; MSC: multipotent mesenchymal stromal cell; SDFT-MSC: tendon-derived MSC; UCM-MSC: umbilical cord matrix-derived MSC).

### *In vitro* differentiation assays

**Figure 5:**
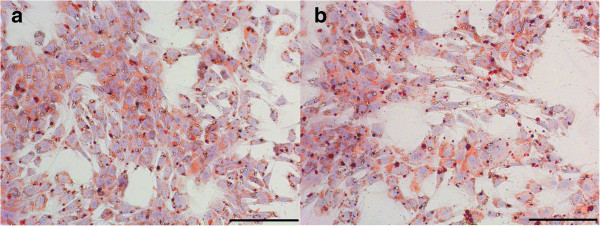
**Adipogenic differentiation.** Adipogenic differentiation demonstrated by the accumulation of intracellular lipid droplets stained by oil red O, shown for MSCs derived from tendon tissue (at 3 days of incubation). **(a)** MSCs isolated by digestion. **(b)** MSCs isolated by explant technique. Scale bar = 200 μm. (MSC: multipotent mesenchymal stromal cell).

Successful induction of adipogenic differentiation was observed in all MSC samples (Figure 5). No distinct differences in the adipogenic differentiation scores were noted between di-MSCs and ex-MSCs, suggesting a similar adipogenic differentiation potential (Table 4).

**Figure 6:**
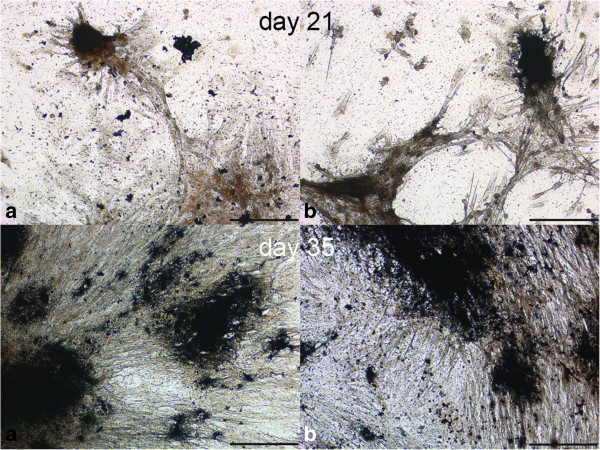
**Osteogenic differentiation.** Osteogenic differentiation with deposition of extracellular calcium visualized by von Kossa staining, shown for MSCs derived from adipose tissue (top: 21 days of incubation; bottom: 35 days of incubation). **(a)** MSCs isolated by digestion. **(b)** MSCs isolated by explant technique. Scale bar = 200 μm. (MSC: multipotent mesenchymal stromal cell).

Extracellular calcium deposits, indicating successful osteogenic differentiation, were observed following von Kossa staining after 21 and 35 days of incubation in all MSC samples derived from AT and SDFT (Figure 6). In di-MSCs and ex-MSCs derived from UCM, one sample in each case did not stain positive for osteogenic differentiation after 21 days of incubation. Following the longer incubation time of 35 days, all UCM-derived ex-MSC samples showed positive von Kossa staining, but one UCM-derived di-MSC sample remained negative. Table 4 provides a summary of IODs of MSCs, measured according to the method of Ostanin et al. (2008) [37]. Interestingly, the IOD for UCM-derived ex-MSCs was lower after 35 days of incubation compared to the IOD at 21 days of incubation.

**Figure 7:**
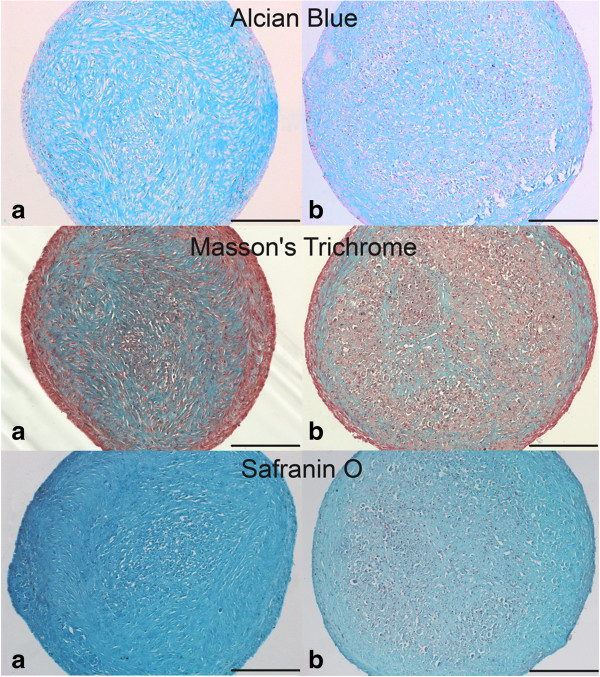
**Chondrogenic differentiation.** Chondrogenic differentiation demonstrated by the presence of glycosaminoglycans and collagen. (top) Alcian Blue, (middle) Masson’s Trichrome and (bottom) Safranin O staining in MSCs derived from umbilical cord matrix after 21 days of incubation. **(a)** MSCs isolated by digestion. **(b)** MSCs isolated by explant technique. Scale bar = 200 μm. (MSC: multipotent mesenchymal stromal cell).

In terms of chondrogenic differentiation, all ex-MSCs, regardless of the tissue type, were positive for glycosaminoglycans and collagen, as demonstrated by Alcian Blue and Masson’s Trichrome staining (Figure 7). Furthermore, all di-MSC samples derived from UCM were able to differentiate towards the chondrogenic lineage. In the case of the di-MSCs derived from AT and SDFT, not all samples showed evidence of chondrogenesis. Two samples from AT and one sample from SDFT did not stain positive for glycosaminoglycans and collagen. Cell pellets that showed successful chondrogenic differentiation, as confirmed by positive Alcian Blue and Masson’s Trichrome staining, were further evaluated using the Bern Score [38] following Safranin O staining (Figure 7). No significant differences were observed between di-MSCs and ex-MSCs (Table 4). Di-MSCs and their corresponding ex-MSCs derived from AT and UCM were assigned similar score points. Ex-MSCs derived from SDFT tended to have slightly higher scores compared to their corresponding di-MSCs.

### Gene expression analysis of tendon markers

**Figure 8:**
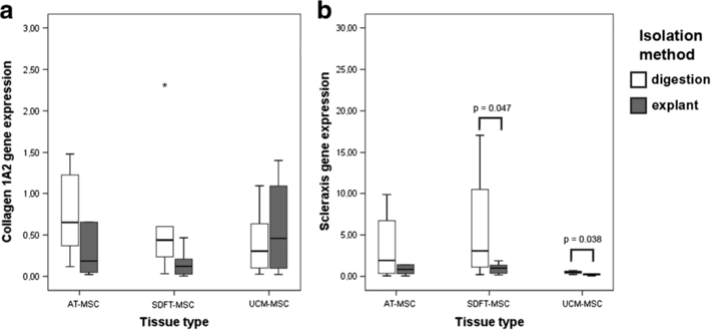
**Expression levels of (a) collagen 1A2 (Col1A2) and (b) scleraxis (Scx).** Star indicates extreme outlier. (AT-MSC: adipose tissue-derived MSC; MSC: multipotent mesenchymal stromal cell; SDFT-MSC: tendon-derived MSC; UCM-MSC: umbilical cord matrix-derived MSC).

A trend towards higher expression of collagen 1A2 was observed in di-MSCs derived from AT and SDFT, in comparison to the corresponding ex-MSCs. In contrast, di-MSCs derived from UCM displayed a trend towards lower gene expression of collagen 1A2, in comparison to their corresponding ex-MSCs (Figure 8a). Di-MSCs showed higher gene expression levels of the tendon marker scleraxis in comparison to ex-MSCs, regardless of the tissue type (Figure 8b). In MSCs derived from SDFT and UCM, the differences were significant, with p-values of 0.047 and 0.038, respectively.

## Discussion

In this study, MSCs were successfully isolated from equine AT, UCM and SDFT by tissue digestion and by explant technique. Further analysis of proliferation, migration and differentiation behaviour of isolated MSCs was performed for comparative evaluation of both MSC isolation methods.

Interestingly, no major differences between cellular properties of di-MSCs and ex-MSCs were observed in the current study, with the exception of a higher level of expression of scleraxis in di-MSCs. However, the tissue digestion method yielded significantly more MSCs in a shorter period of time.

For this study, horses were chosen as donor animals, as the treatment of orthopaedic injuries with MSCs is currently a widely used treatment in equine medicine [3-5]. Furthermore, due to pathophysiological similarities between human and equine orthopaedic diseases, the horse is an appropriate model for orthopaedic research in human medicine [39].

Three different solid tissues, AT, SDFT and UCM, were chosen for exemplary evaluation of MSC isolation methods. These tissues were considered suitable as they were already known to host MSCs [15,16,18] and also because their different densities and stiffness allowed for evaluation of protocols under different conditions.

Different techniques exist for isolation of MSCs from diverse sources. In this study, both a standard tissue digestion using collagenase and MSC isolation by explant technique were performed. Enzymatic digestion by collagenase, first described by Rodbell (1964) [40], is a widely used method for degradation of the collagen network of solid tissue. Nonetheless, some studies have described disadvantages to this method, such as the relatively high costs of reagents, time-consuming labour and inconsistent results due to heterogeneous preparations of pure collagenase solutions [41-44]. To avoid the latter, a uniform batch of collagenase has been used in this study.

Previous studies have investigated other enzymatic methods for MSC isolation, such as the use of liberase, trypsin and hyaluronidase, in order to achieve a reproducible and qualitatively improved tissue digestion and avoid damage to the isolated cells, as an alternative to crude collagenase digestion [20,41,45,46]. However, the use of these alternative enzymatic methods is not without controversy [47]. Therefore, in the present study, standard enzymatic digestion using collagenase was performed for the comparison of MSC isolation techniques. In order to achieve the mildest enzymatic treatment, collagenase concentrations and incubation times were adapted to the requirements of the tissues used as MSC sources, which had been evaluated earlier (unpublished data), as previously suggested by others [30,47-49].

Several studies have reported the effects of enzymes, endotoxin and chemotactic tissue breakdown products on the phenotype and behaviour of cells [30,41,47,50], [51]. Therefore, we considered a non-enzymatic isolation technique to recover MSCs, which would potentially be less affected and damaged than by an enzymatic isolation technique [28,52,53]. In this study, the isolation of MSCs by explant technique was performed and compared to collagenase digestion. The obvious benefit of this non-enzymatic cell isolation technique is that the procedure is simpler in comparison to the enzymatic method and does not require expensive enzymes.

We hypothesized that collagenase digestion affects the isolated cells, while the explant technique does not, thus resulting in differences between the cellular properties of di-MSCs and ex-MSCs. However, this hypothesis was not supported by the present study, as no major differences were found regarding most cellular properties investigated here.

The most important difference between the two MSC isolation techniques was that collagenase digestion yielded significantly more MSCs than the explant technique, which is consistent with published data [28,44,47]. A possible explanation might be that only cells located at the tissue margin can migrate out of the tissue in the explant technique, so that not all MSCs residing in the tissue can be collected when using this technique. A practical option to improve the MSC yield from explant cultures might be to minimize the size of the tissue samples. The MSC numbers per gram of tissue that were obtained by each isolation method in this study are within a similar range to MSC yields reported in other studies [21,28]. Despite significant differences between cell yields, which might suggest differences between the isolated cell populations, di-MSCs and ex-MSCs displayed similar characteristics during further analyses.

Variation in isolation protocols, as well as alteration of culture conditions, have been reported to influence proliferation of MSCs [31,32,34]. This could be due to the fact that some extrinsic substances may be toxic and induce cell death [31,32] and, therefore, cause variation in viability and expansion potential. In the present study, di-MSCs required less time for primary cultivation in comparison to ex-MSCs. It is likely that this effect was due to the fact that digestion initially makes more cells accessible, rather than to differences in proliferation potential of di-MSCs. In all subsequent passages, when the initial seeding density was standardised, proliferation of di-MSCs and ex-MSCs was similar.

Migration potential of MSCs is considered important for their integration into the host tissue during therapeutic applications. Several studies have reported inhibition or increase of migration capacity by different drugs *in vitro*[31,54,55]. Furthermore, a comparative study of different protocols for isolation of BM-MSCs also showed that isolation conditions affect migration ability [34]. Similarly, the technique used to isolate MSCs may affect the ability of MSCs to migrate from solid tissues. In the present study, di-MSCs derived from all the tissues studied, showed a trend towards increased migration activity in comparison to ex-MSCs. This finding was surprising, given that the ex-MSCs had initially been selected based on their migratory capacity during the isolation procedure. Further studies to investigate these effects are necessary. Furthermore, the cultivation of MSCs in a 3D-spheroid assay may be advantageous for injection of MSCs. The investigation of migration potential in these spheroids could potentially be used to assess the ability of the applied MSCs to leave the spheroids and migrate into surrounding host tissue.

In this study, no significant differences were observed in the adipogenic, osteogenic or chondrogenic differentiation capacity between di-MSCs and ex-MSCs. This finding is consistent with results of previous studies in which different methods for isolation of MSCs were compared [28,34]. In contrast, several studies have demonstrated that changes in culture conditions or cultivation alone seem to affect cellular properties such as the differentiation potential [31,51,56]. Due to standardised and optimised differentiation conditions, such influences on differentiation potential were avoided in the present study.

Analyses of osteogenic and chondrogenic differentiation were performed using standard protocols described in the literature [57]. The observed decrease in IOD for UCM-derived ex-MSCs following longer incubation could be due to partial cell detachment during the longer cultivation time.

The adipogenic differentiation protocol was modified based on an evaluation of different protocols for equine MSCs [36], as insufficient adipogenic differentiation of equine MSCs has been repeatedly reported after standard induction [26,58]. The modification of the protocol included supplementation of adipogenic differentiation medium with rabbit serum, of which the benefit for adipogenic differentiation has been previously described [26,36,58].

Significantly higher expression levels of the tendon phenotype-related gene scleraxis [59-62], were found in MSCs isolated by enzymatic digestion. Alterations in expression levels of other target genes following the digestion technique have been shown in several studies [51,56,63]. Potentially, these alterations are caused by the altered environmental conditions the cells are subjected to during enzymatic tissue digestion. It could be also hypothesised that collagen breakdown products from the digested tissues trigger upregulation of the scleraxis gene. There were no noticeable differences in expression levels of collagen 1A2, one of the key components of tendon matrix [64,65], between di-MSCs and ex-MSCs. It is possible that collagen expression is more stable to influence by extrinsic factors. This hypothesis is supported by published data showing that no variations in transcription level of collagen were observed following supplementation of cell culture medium with ibuprofen [33]. However, only the expression of collagen 1A2 was investigated in present study. During tendon degeneration, initially there is an increased level of collagen 3 fibers which are probably only later replaced by collagen 1 fibers [65]. Hypothesising that collagen breakdown products produced during tissue digestion simulate the early phase of healing and thus stimulate upregulation of tendon markers, collagen 3 expression might be upregulated rather than collagen 1 expression. Still, whether a higher expression of tendon markers *in vitro* reflects the situation *in vivo* and is a reliable indicator of the effect of MSCs on tendon healing remains to be evaluated.

The cell population harvested by enzymatic digestion is potentially heterogeneous, and this raises the question as to whether the isolated cells are in fact MSCs. Further evaluation of cellular properties is important to determine whether these cells represent tissue specific cells, such as tenocytes, or display characteristics of MSCs [33,59,64].

Cells isolated in this study were identified as MSCs based on their capacities for self-renewal, plastic-adherence and tri-lineage differentiation. These characteristics are regarded as minimal, but adequate, criteria for identification of MSCs [26,34,66].

Evaluation of expression of specific stem markers would have provided more accurate information. However, in contrast to human cells, an established set of equine MSC-specific cell surface markers is not yet available due to the limited reactivity of available antibodies with equine epitopes [67,68].

Despite the lack of evaluation of MSC markers, the results of this study show that the isolation method has no major influence on cellular growth and tri-lineage differentiation characteristics. Furthermore, no negative effects of collagenase digestion on the isolated cells were observed. Our results are in accordance with the suggestion that alterations in experimental conditions are of minor importance to cell behaviour in comparison to cell source and interdonor variability [20]. Nevertheless, optimisation and standardisation of isolation protocols are required in order to improve comparability of results obtained in different studies [21,35].

## Conclusions

Collagenase digestion and the explant method are both feasible and effective techniques for isolation of MSCs from solid tissues. In this study, the MSCs obtained via both methods displayed similar growth characteristics and tri-lineage differentiation capacities. However, isolation of MSCs from solid tissues by digestion appears advantageous for therapeutic use due to the higher obtainable MSC yields with less time in primary culture. Furthermore, the higher gene expression levels of scleraxis in di-MSCs suggest a potentially more effective role for these cells in tendon regeneration. Further investigation to confirm this hypothesis is required.

## Abbreviations

AT: Adipose tissue; AT-MSC: Adipose tissue-derived MSC; BM: Bone marrow; BM-MSC: Bone marrow-derived MSC; Col1A2: Collagen 1A2; di-MSC: MSC isolated by digestion method; DMEM: Dulbecco’s modified Eagle medium; ex-MSC: MSC isolated by explant technique; FCS: Foetal calf serum; GADPH: Glyceraldehyde 3-phosphate dehydrogenase; GT: Generation time; HBSS: Hank’s balanced salt solution; IOD: Index of osteogenic differentiation; IQR: Interquartile range; MNC: Mononuclear cell; MSC: Multipotent mesenchymal stromal cell; MTS: 3-(4,5-dimethylthiazol-2-yl)-5-(3-carboxymethoxyphenyl)-2-(4-sulfophenyl)-2H-tetrazolium; P: Passage; PBS: Phosphate-buffered saline; PR: Proliferation rate; qPCR: Real-time quantitative PCR; Scx: Scleraxis; SDFT: Superficial digital flexor tendon; SDFT-MSC: Tendon-derived MSC; UCM: Umbilical cord matrix; UCM-MSC: Umbilical cord matrix-derived MSC.

## Competing interests

The authors declare that they have no competing interests.

## Authors’ contributions

CG designed the study, collected and processed the specimens, assembled and analysed the data and drafted the manuscript. WB supervised the study and helped with editing and revision of the manuscript. JB participated in the design of the study, sample collection and processing, contributed to data analysis and interpretation and helped with editing and revision of the manuscript. HJ contributed to the design of the study and data interpretation. CS provided technical and scientific advice. IR helped in the design of the study, sample collection and processing, contributed to data interpretation and helped with editing and revision of the manuscript. All authors read and approved the final manuscript.
